# 10-year mortality, causes of death and cardiovascular comorbidities in idiopathic normal pressure hydrocephalus

**DOI:** 10.1007/s00415-023-12067-5

**Published:** 2023-11-02

**Authors:** Sanna A. Eklund, Hanna Israelsson, Mattias Brunström, Karin Forsberg, Jan Malm

**Affiliations:** 1 Department of Clinical Science, Neurosciences, Umeå University, Umeå, Sweden; 2 Department of Health, Medicine and Caring Sciences, Linköping University, Linköping, Sweden; 3 Department of Public Health and Clinical Medicine, Umeå University, Umeå, Sweden

**Keywords:** Normal pressure hydrocephalus, Mortality, Cardiovascular disease, Comorbidities, Causes of death

## Abstract

**Objective:**

The objective was to investigate 10-year mortality, causes of death and cardiovascular comorbidity in idiopathic normal pressure hydrocephalus (iNPH) and to evaluate their mutual associations.

**Methods:**

This prospective cohort study included 176 CSF-shunted iNPH patients, and 368 age- and sex-matched controls. At inclusion, participants were medically examined, had blood analyzed and answered a questionnaire. The vascular comorbidities investigated were smoking, diabetes, body mass index, blood pressure (BP), hyperlipidemia, kidney function, atrial fibrillation and, cerebro- and cardiovascular disease.

**Results:**

Survival was observed for a mean period of 10.3 ± 0.84 years. Shunted iNPH patients had an increased risk of death compared to controls (hazard ratio (HR) = 2.5, 95% CI 1.86–3.36; *p* < 0.001). After 10 years, 50% (*n* = 88) of iNPH patients and 24% (*n* = 88) of the controls were dead (*p* < 0.001). The risk of dying from cardiovascular disease, falls and neurological diseases were higher in iNPH (*p* < 0.05). The most common cause of death in iNPH was cardiovascular diseases (14% vs 7% for controls). Seven out of nine iNPH dying from falls had subdural hematomas. Systolic BP (HR = 0.985 95% CI 0.972–0.997, *p* = 0.018), atrial fibrillation (HR = 2.652, 95% CI 1.506–4.872, *p* < 0.001) and creatinine (HR = 1.018, 95% CI 1.010–1.027, *p* < 0.001) were independently associated with mortality for iNPH.

**Discussion:**

This long-term and population-matched cohort study indicates that in spite of CSF-shunt treatment, iNPH has shorter life expectancy. It may be important to treat iNPH in supplementary ways to reduce mortality. Both cardiovascular comorbidities and lethal falls are contributing to the excess mortality in iNPH and reducing these preventable risks should be an established part of the treatment plan.

**Supplementary Information:**

The online version contains supplementary material available at 10.1007/s00415-023-12067-5.

## Introduction

Idiopathic normal pressure hydrocephalus (iNPH) is a neurodegenerative syndrome characterized by gait disturbance, dementia and urinary incontinence. It is common in the elderly population and can be partially treatable by a CSF shunt. iNPH has an increased risk of all-cause mortality, hazard ratio 1.8–3.8, compared to the population based on observation times between 3 and 11 years [1–3]. Studies show that there is a high prevalence of vascular comorbidity [4–6] which may not effect long-term surgery outcome [7] but still can result in premature death [1, 5, 8, 9]. The specific vascular comorbidities arterial hypertension, diabetes mellitus, coronary heart disease, hyperlipidemia and obesity are associated with iNPH [4, 10–13]. Cerebrovascular and cardiovascular diseases are overrepresented causes of death [1, 2, 14]. The association between specific vascular risk factors (VRF) and mortality have been systematically investigated in Alzheimer’s Disease, vascular dementia and Lewy Body dementia [15–17]. While in iNPH which specific VRF that are most important for mortality remain to be determined [1, 5, 8].

Intensive treatment of specific VRF is important for reducing morbidity and mortality in the population as a whole [18–20] and should be valid in iNPH too because of the high vascular comorbidity revealed in this patient group. We hypothesize that by identifying the most important VRF related to death in iNPH, prospective intervention studies can be designed to confirm effect of treatment. The objective of this prospective iNPH cohort study with 10-year follow-up of mortality was to compare long-term mortality and causes of death in iNPH to the normal population and to identify which specific VRF are associated to mortality in iNPH.

## Methods

### Summary

In this prospective cohort study, 176 shunted iNPH patients and 368 age- and sex-matched controls from the population were included. At baseline, participants were examined by a physician and answered a questionnaire regarding VRF. Mortality was then observed starting at date of shunt surgery. After a mean of 10.3 years observation, data on time and cause of death were collected.

### Study population

**Fig. 1:**
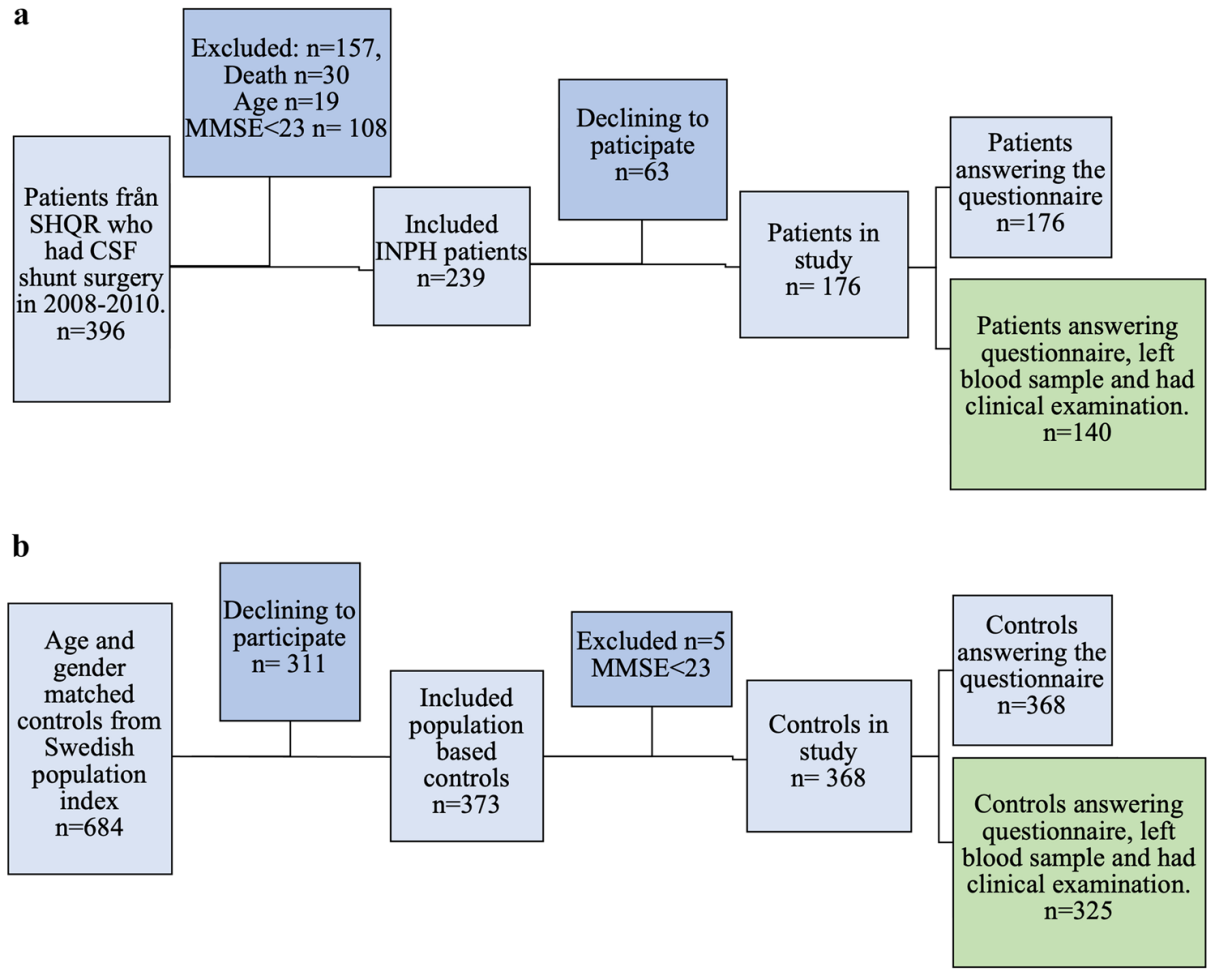
**a** Flow chart of patient selection. **b** Flow chart of control selection. SHQR, Swedish Hydrocephalus Quality Registry; MMSE, Mini-Mental State Examination

In Sweden, all patients who undergo CSF-shunt surgery for iNPH are registered in the Swedish Hydrocephalus Quality Registry (SHQR). Using SHQR, all iNPH patients that had CSF-shunt surgery between the years 2008 and 2011 were invited to participate in the present study. Exclusion criteria were: MMSE < 23 points, death before study start, or age below 60 years or above 80. The study included 176 patients (41.5% women, mean age 73.7 years). See flowchart of participant selection in Fig. 1a.

From the Sweden Population Index, four age and sex-matched controls for each patient were recruited. Age match was done by including the four individuals born on the dates closest to each included patient and living in the Umeå municipality. Exclusion criteria were the same as for patients. A total of 368 controls were included in the study (36.7% women, mean age 72.8 years). See flowchart in Fig. 1b.

Clinical characteristics of patients and controls are displayed in Table 1. There were no significant differences between patients and controls regarding age, sex, and mortality observation time.

**Table 1:** Characteristics for patients and controls

	Patients	Controls	*p* value (*t*-test)
*n*	176	368	–
Age ± SD	74 ± 6.0	73 ± 5.8	0.06
Sex, females	73 (41%)	135 (37%)	0.28
FRS ± SD	27.8 ± 15.0	22.0 ± 13.6	< 0.001
Mean survival	8.6 ± 2.4	9.6 ± 1.9	< 0.001
Mean observation time mortality	10.3 ± 0.86	10.3 ± 0.84	0.5
Median observation time mortality (range)	10.3 (9.1–12.0)	10.2 (9.1–12.0)	0.5
*n* (%) deaths during observation time	88 (50%)	88 (24%)	< 0.001

FRS, Framingham risk score; SD, standard deviation

### Vascular risk factors

Patients and controls had a physical examination including ECG, blood sample, weight, length, and blood pressure and were asked to answer a questionnaire at start of study when patients and controls were included. The individual risk factors investigated were age, smoking, systolic blood pressure (SBP), lipids, body mass index, atrial fibrillation, diabetes, and kidney function.

Framingham risk score (FRS) was calculated according to its manual [21]. An algorithm was used for converting ApoA1 and ApoB to total cholesterol and HDL [22] and has been previously described [9].

### Mortality

Between the set years 2009–2019, date of death and causes of death were obtained from the Swedish Board of Health and Welfare. Start of observation was date of CSF-shunt operation for iNPH patients. For controls, start was the same date as the operation date of their matched patient. When a person deceases in Sweden, a death certificate is made by the responsible physician and death cause is labeled with one “underlying cause” and often several “contributing causes”. ICD-10 codes of underlying cause of death were summarized into nine broader categories based on the A-Z chapters of ICD-10 [23]. The categories and ICD-10 codes are: Infectious disease (A, B, J00–J22, N39), Neoplasms and diseases of blood (C, D), Dementias (F00–F03, G30), Neurological disease (G excl. G30), Cerebrovascular disease (I60–I69), Diseases of the circulatory system (I excl. I60–I69), Respiratory system (J excl J00–J22), External cause (V, W, X, Y) and Other (F excl F00-03, K, L, M, N, Q, R). Investigating increased risk of selected causes of death between patients and controls were done by creating binary variables for each specific cause, based on both underlying and contributing diagnoses listed on the death certificate. The four causes especially investigated were: falls (W00–Wi19, X59), subdural hematoma (SDH) (S06.5, I62.0), neurological diseases and dementia (G00–G99, F00–F03) and diseases of the cardiovascular system (I00–I99). iNPH was reviewed for shunt-related causes of death, also using underlying and contributing causes (G00.0–G00.9, G97.2, T85.8).

### Statistical analysis

Statistical significance level was set to *p* < 0.05. SPSS version 28.0.1.1 was used for statistical analysis. Independent sample *t*-test was used for characteristic comparison between the groups, and the number of deaths during follow-up was analyzed with Chi-square. COX regression analysis was used for determining association between survival and risk factors and for survival comparison between patients and controls. Time to event was calculated from shunt surgery date for patients and matched hypothetical date for controls to date of death. The individual risk factors were analyzed both unadjusted and adjusted for age and sex. An interaction variable between VRF and group (iNPH vs controls) was analyzed with COX regression, unadjusted and adjusted for age and sex. Covariates were chosen to further minimize potential confounders that could not be diminished through study design. All causes of death were presented descriptively and for comparison of risk for dying because of a specific cause, COX regression analysis and Kaplan–Meier method were used. Participants who had died within the observation time, but from another cause than the specific investigated cause, were censored in the Kaplan–Meier analysis.

### Ethical standards

The study was approved by the Institutional Review Board at Umeå University and is registered at clinicaltrials.gov (NCT01850914). Oral and written informed consent according to the Declaration of Helsinki was obtained. To ensure that participants could give a reliable informed consent, the MMSE limit was set to a score of > 23 points to be included in the study.

## Results

### Mortality

**Fig. 2:**
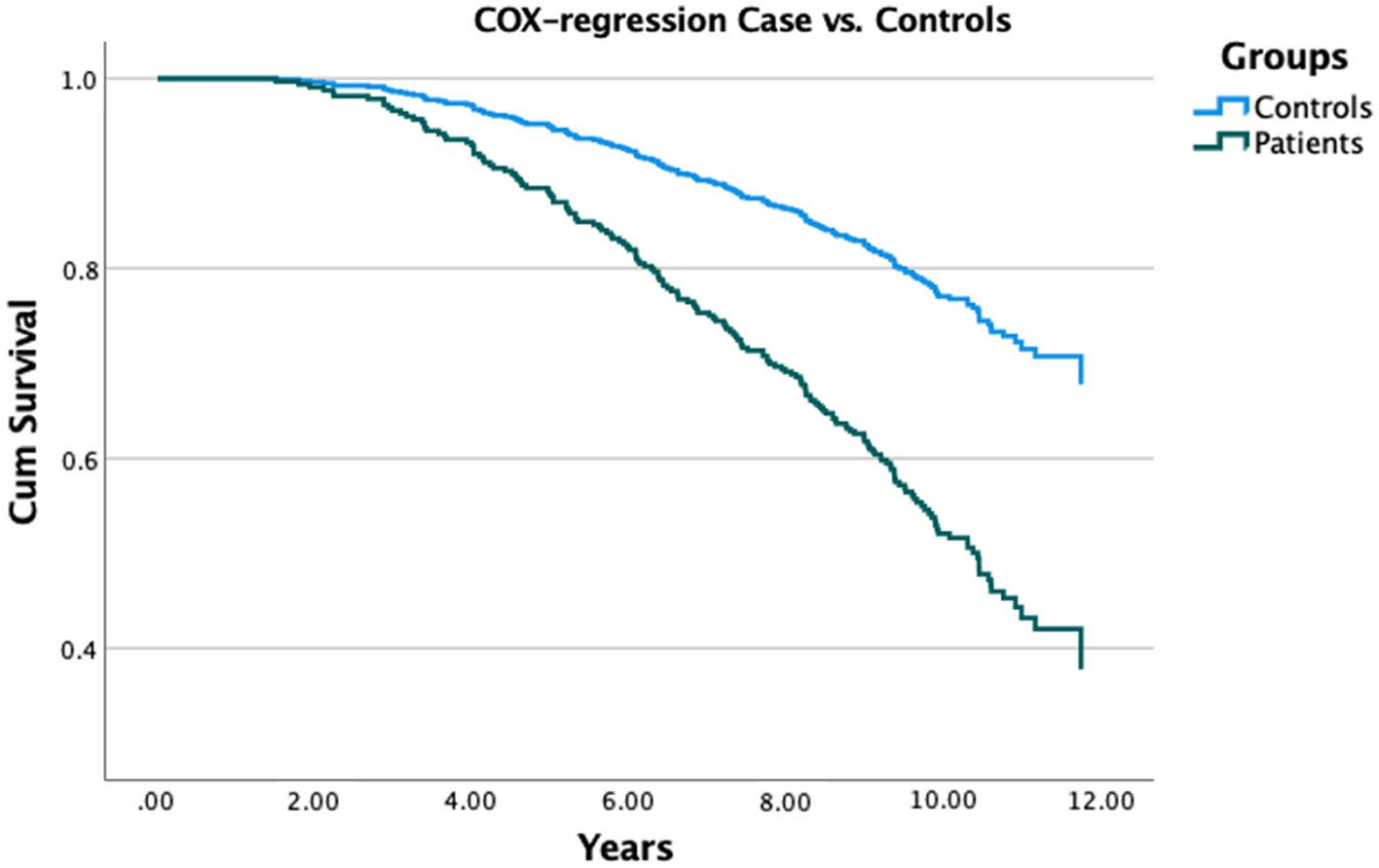
Plot of COX regression analysis between patients and controls, p < 0.001

Mean observation time for patients and controls were 10.3 ± 0.85 years (± SD), respectively. After follow-up time, 88 of 176 (50%) of iNPH and 88 of 368 (24%) controls had died (*p* < 0.001). iNPH patients had an increased risk for mortality compared to controls (hazard ratio (HR) = 2.5, 95% CI 1.86–3.36, *p* value < 0.001). See survival plot in Fig. 2. When adjusted for total vascular comorbidity using Framingham risk score, the difference between patients and controls remained significant (HR = 2.7, 95% CI 1.9–3.9, *p* < 0.001).

### Causes of death

**Fig. 3:**
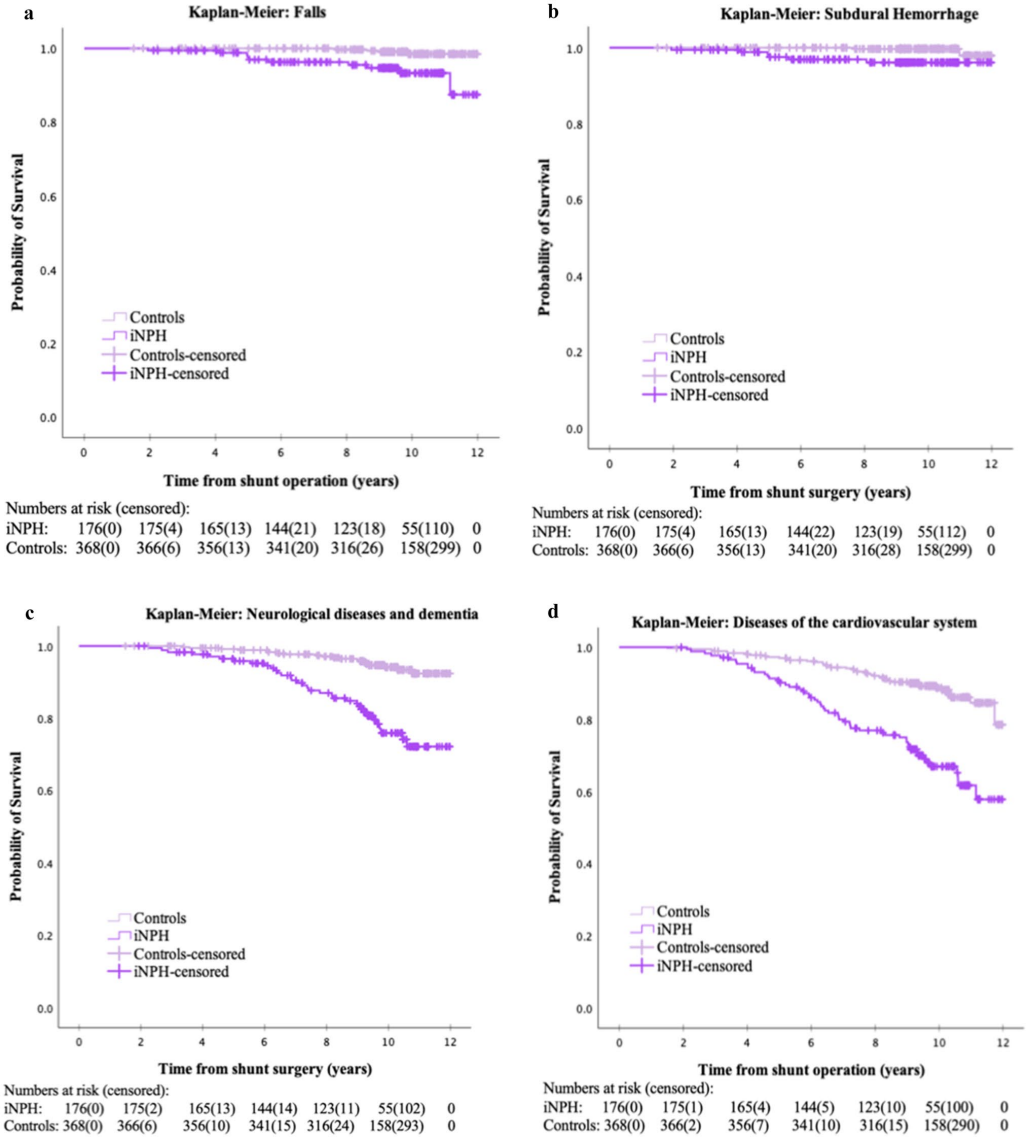
Kaplan–Meier plots displaying the increased risk of dying of a specific cause of death, iNPH compared to controls. Other causes of death within observation time or at end of observation time were marked as censored. Analyzed with log-rank test. **a** Plot of falls, *p* < 0.001. **b** Plot of subdural hemorrhage, *p* = 0.004. **c** Plot of neurological diseases and dementia, *p* < 0.001. **d** Plot of diseases of the circulatory system, *p* < 0.001

**Fig. 4:**
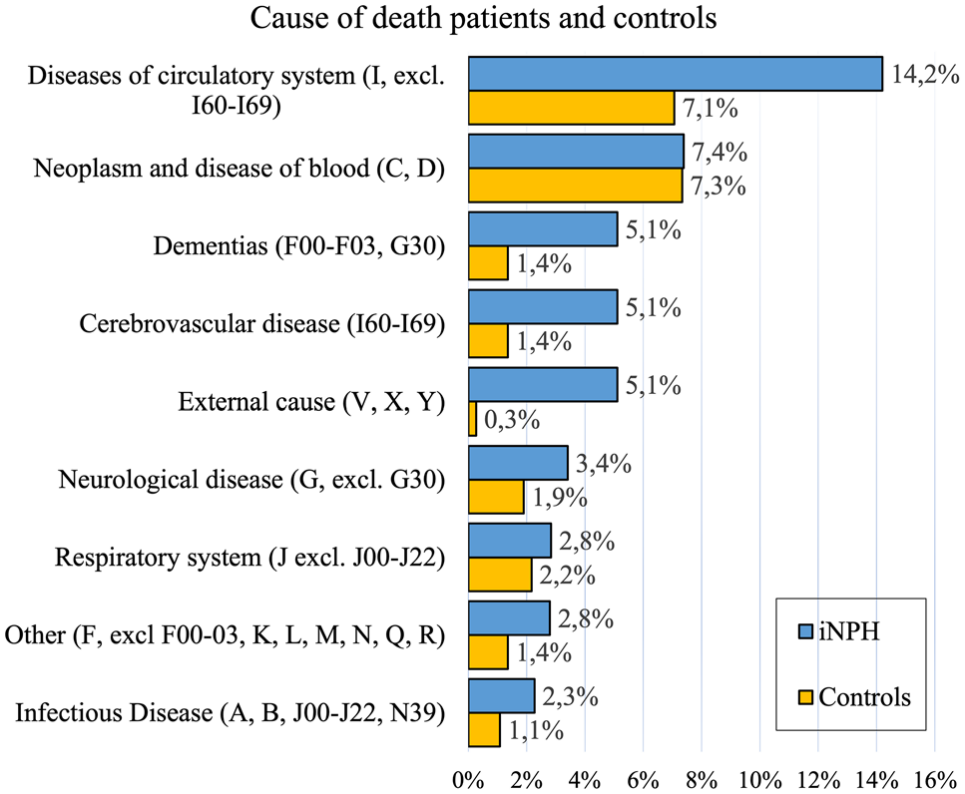
Distribution of underlying causes of death for iNPH and controls. Diagnosis codes of each category within brackets

The risk of dying from cardiovascular disease (*p* < 0.001), falls (*p* < 0.001), subdural hematoma (*p* = 0.004) and neurological diseases (*p* < 0.001) were higher for patients than controls (Table 2). Kaplan–Meier plots for all four causes of death are displayed in Fig. 3. The most common cause of death for patients was cardiovascular diseases (14.2% versus 7.1% of the controls). Nine patients versus one control had external cause listed as underlying cause of death. Four of those patients had falls and the remaining five were registered as “Exposure to unspecified factor causing injury”. Seven out of the nine patients with external cause had subdural hematoma (SDH) listed as contributing cause of death. Six of the seven SDH were traumatic (acute) and one was non-traumatic but not specified acute or chronic. No shunt dysfunction, bacterial meningitis or hygroma was listed as cause of death in iNPH. See Fig. 4 for full distribution of underlying causes of deaths.

**Table 2:** COX regression of cause of death with patients and controls as covariate

Cause of death	Deaths (*n*) (iNPH + controls)	COX regression analysis, HR (95% CI)	*p* value	Kaplan–Meier method, log-rank test
Falls (W00–W19, X59)	14	6.4 (2.0–20.4)	0.002	< 0.001
Subdural hemorrhage (S06.5, I62.0)	8	7.3 (1.5–36.5)	0.015	0.004
Diseases of cardiovascular system (I00–I99)	100	3.1 (2.1–4.7)	< 0.001	< 0.001
Neurological disorders and dementia (G00–G99, F00–F03)	54	4.3 (2.5–6.5)	< 0.001	< 0.001

Kaplan–Meier methods and log-rank test of the four specific causes of death comparing patients and controls. HR, hazard ratio; CI, confidence interval. Cause of death with ICD code within brackets

### Vascular risk factors

Univariable COX regression analysis with patients and individual risk factors showed that: age (HR = 1.074, 95% CI 1.035–1.114, *p* < 0.001), female sex (HR = 0.582, 95% CI 0.373–0.902, *p* = 0.017), SBP (HR = 0.985, 95% CI 0.972–0.997, *p* = 0.018), atrial fibrillation (HR = 2.652, 95% CI 1.506–4.872, *p* < 0.001) and creatinine (HR = 1.018, 95% CI 1.010–1.027, *p* < 0.001) were associated with mortality. Framingham risk score was also associated with mortality for patients (HR = 1.019, 95% CI 1.003–1.035, *p* = 0.021). Systolic blood pressure (HR = 0.986, 95% CI 0.973–1.0, *p* = 0.044), atrial fibrillation (HR = 2.473, 95% CI 1.4–4.37, *p* = 0.002) and creatinine (HR = 1.013, 95% CI 1.003–1.023, *p* = 0.001) remained associated to mortality after adjustment for age and sex. Interaction models with individual VRF and group (iNPH vs. control) were not significant. See Table 3 for analyses of VRF with patients, both unadjusted and adjusted for age and sex. Analyses on VRF and controls, the total population and p value for interaction models are presented in eAppendix Table 1 and eAppendix Table 2.

**Table 3:** Univariable COX regression analysis with patients and individual risk factors

	Patients	Univariable COX regression, unadjusted			Univariable COX regression, adjusted for age and sex		
	*N*	HR	95% CI	*p* value	HR	95% CI	*p* value
Smoking	172	1.057	0.688–1.623	0.801	1.193	0.77–1.85	0.43
BMI	138	1.029	0.983–1.077	0.227	1.041	0.995–1.09	0.079
Diabetes diagnosis	142	1.115	0.649–1.913	0.694	0.845	0.483–1.476	0.553
P-Glucose	138	1.015	0.912–1.13	0.788	0.959	0.857–1.074	0.47
Systolic blood pressure	140	0.985	0.972–0.997	0.018*	0.986	0.973–1.0	0.044*
Age	176	1.074	1.035–1.114	< 0.001*	N/A	N/A	N/A
Sex (female)	176	0.582	0.373–0.902	0.017*	N/A	N/A	N/A
Atrial fibrillation	166	2.652	1.506–4.872	< 0.001*	2.473	1.4–4.37	0.002*
ApoB	140	1.001	1.00–1.001	0.25	1.001	1.00–1.002	0.162
ApoA1	140	0.999	0.998–1.00	0.135	1.0	0.999–1.001	0.813
ApoB/ApoA1	140	2.427	0.981–6.005	0.055	1.832	0.738–4.55	0.192
Hyperlipidemia yes/no	143	1.044	0.644–1.690	0.862	1.102	0.679–1.788	0.694
Creatinine	140	1.018	1.010–1.027	< 0.001*	1.013	1.003–1.023	0.01*
FRS	134	1.019	1.003–1.035	0.021*	N/A		

Unadjusted and adjusted for age and sex. Significant results are marked with *. BMI, body mass index; FRS, Framingham risk score; HR, hazard ratio; CI, confidence interval; VRF, vascular risk factors; N/A, not applicable

## Discussion

This long-term mortality study with 176 iNPH patients investigating the influence of VRF on survival showed that iNPH have a substantially higher risk of death within 10 years compared to matched controls. The risk of deadly falls, subdural hematoma and cardiovascular death were increased for iNPH. Low systolic blood pressure, atrial fibrillation and worse kidney function decreased survival.

Falls as a cause of death is 17 times more likely among iNPH than controls which is an important finding since it is a highly preventable cause of death. A cardinal feature of iNPH is gait disturbance [24], subsequently, falls are very common [25]. Of those who died from falls in this study, several had subdural hematoma. It is possible that the increased vulnerability for SDH in iNPH could be because of anti-coagulant treatment or shunt over-drainage. This is something treating physicians should be aware of and the authors suggest further research investigating the causality between falls, subdural hematoma, anti-coagulants and shunt dysfunction. Adequate measures should be taken to prevent falls for iNPH similar to the standards of older adults with gait disturbances [26–28]. For stroke patients with balance and gait sequelae, the outpatient care is comprehensive and organized by an interprofessional team including physiotherapist, occupational therapists and social services to prevent dangerous falls [29]. To delay death in iNPH, the fall risk needs to be considered and treated. This should probably be managed with a comprehensive approach and in cooperation with other categories of health professionals and specialities.

After 10 years of observation, more than double had died in the iNPH group compared to the controls even though the mean survival did not differ considerably between the groups. This could indicate that many patients die in the later part on the observation time and for a better understanding of the mortality in iNPH a longer follow-up is essential. The hazard ratio for death in this study is in the middle of the previously showed span in iNPH [1–3]. The present study contains a relatively long follow-up time and, in comparison, a large study sample which could be reason to consider the present result more precise than previous studies.

This study showed SBP, atrial fibrillation and kidney function to be important predictive individual risk factors for survival. Kidney function has an established association to mortality and low function leads to increased risk of cardiovascular death [30]. This study supports that this is accurate for iNPH patients too. Low SBP was associated to increased mortality which is surprising with regard to previous studies showing that high SBP is associated with iNPH. The hypothesis behind this controversial finding is multifactorial. First, it is known that low blood pressure’s association to higher mortality is a recurrent phenomenon in frail and elderly patients [31–33]. The SBP association could, likewise, be an indicator of the frailty of iNPH patients. Second, the finding could also be explained by hypoperfusion of vital organs such as kidneys and the heart in persons with low blood pressure, increasing the risk of death [34, 35] or speak to other autonomic neuropathological mechanisms that increases the risk of death, for example, orthostatism [36] leading to syncope and falls. Associated risk factors in this study diverges from other iNPH studies where diabetes type 2, atrial fibrillation, heart disease and previous stroke are associated [1, 5]. An explanation to the differences in results could be that previous studies have investigated the prevalence of vascular comorbidities and their association to survival based on retrospective registry data and ICD-codes from medical records [1, 5, 8]. Several VRF are scales, not dichotomous comorbidities, and should be evaluated as such. This study probably provides more accurately asserted evidence of VRF since it uses prospective measured values. Even though the separate analysis of VRF and iNPH are significant, the interaction models including group in this study were not. This result should be interpreted with caution because of the small cohort. It can, however, indicate that there are no specific VRF that are especially deadly for iNPH compared to controls and that the treatment plan for VRF in iNPH should be the same as for the population. Previous studies have not reported interaction models of VRF and group and thus our results cannot be compared [1, 5]. The separate analysis of VRF is nevertheless important because it can be predictive and speak to contributing factors to the cause of death. In addition, accumulated risk factors and diseases may act synergistically. For example, the combination of a CSF-shunted iNPH patient with gait disturbances, atrial fibrillation and anti-coagulant medication could hypothetically make a fall more common and head trauma more deadly.

Interestingly, when adjusting the survival for Framingham risk score, which is a combined score for vascular comorbidity, iNPH still had an increased risk of mortality. This indicates that the high vascular comorbidity this patient group usually have cannot alone explain the excess mortality. It is possible that iNPH and its manifestations independently affect survival too. There are reasons to why physicians should treat VRF even though iNPH seemingly die irrespectively of cardiovascular comorbidities. One reason is because high VRF also contribute to cardiovascular disease which is a major cause of death in iNPH. Patients with myocardial infarction have an increased mortality, possibly because of damage to the heart or the fragility of the patient group. Regardless, they receive VRF treatment to reduce the risk for additional infarcts. If high VRF contribute to the progress of iNPH then treating the VRF in iNPH would be motivated with the same argument as for myocardial infarct patients, to prevent additional progress of the disease.

The most prominent strength of this prospective cohort study compared to preceding iNPH mortality studies is the combination of long observations time, the relatively large iNPH cohort and the fact that the VRF are clinically assessed in real-time. Evaluating the VRF using clinical data, collected with the same protocol for both patients and controls, gives this study a unique material for investigating the association between VRF and mortality. Another strength is the large and thoroughly age and gender matched control group from the population which elevates the accuracy of the survival analysis.

One limitation is that the death causes data comes from those reported on death certificates. This data is not always reliable since it is performed by individual physicians and sometimes without complete vigilance. For example, unexpectedly few of iNPH were reported to have either dementia or iNPH as a main cause of death which is in accordance with previous observations regarding the underreporting of dementias on death certificates [37]. However, the same method was used on all participants and the Swedish Board of Health and Welfare uses a systematical method based WHO’s international framework [38] for deciding the underlying cause of death [39]. Hence, it is the best available data on cause of death but one can expect that dementias and iNPH are more often contributing to death than the rate presented in this study. When adjusting the COX regression model for Framingham risk score there might be built in attrition bias in the model because some iNPH were excluded for missing values when calculating FRS. Thus, one should compare the unadjusted HR and FRS-adjusted HR with caution because they are based on slightly different cohorts of iNPH patients. Data on atrial fibrillation were based on self-reported data from questionnaires and can lead to information bias, both increasing and decreasing the reported cases. Since the same collecting method is used for patients and controls, the risk of skewing of the results is reduced.

In conclusion, in spite of CSF-shunt treatment, iNPH have a higher mortality than a matched population. Partially preventable causes such as lethal falls and cardiovascular comorbidities are contributing to shorter life expectancy, highlighting why these risk factors should be systematically assessed and treated in iNPH. By implementing protocols for preventing falls and treating cardiovascular comorbidities utilizing other healthcare professions, excess mortality could probably be decreased in iNPH. Intervention studies in the subject are needed.

### Supplementary Information

Below is the link to the electronic supplementary material.

Supplementary file1 (DOCX 19 kb)
