# Supplementary material for: 10-year mortality, causes of death and cardiovascular comorbidities in idiopathic normal pressure hydrocephalus
Source: J Neurol. 2023 Nov 2;271(3):1311–9. doi: 10.1007/s00415-023-12067-5 (PMC10896765; doi:10.1007/s00415-023-12067-5)
Supplement: Supplementary file 1 — Supplementary file1 (DOCX 19 kb) [file 415_2023_12067_MOESM1_ESM.docx]

| **Table 1** | **Controls** | **Univariable COX-regression, unadjusted** | | | **COX regression for total population + p-value for interaction variable (VRF*group)** | | | |
| --- | --- | --- | --- | --- | --- | --- | --- | --- |
|  | N | HR | 95 % CI | p-value | HR | 95 % CI | p-value | p-value interaction variable |
| **Smoking** | 365 | 1.445 | 0.939-2.222 | 0.094 | 1.448 | 0.941-2.226 | 0.092 | 0.306 |
| **BMI** | 325 | 0.988 | 0.929-1.052 | 0.702 | 0.989 | 0.929-1.052 | 0.717 | 0.3 |
| **Diabetes diagnosis** | 337 | 2.025 | 1.158-3.541 | 0.013* | 2.006 | 1.148-3.506 | 0.015* | 0.135 |
| **P-Glucose** | 325 | 1.127 | 1.018-1.249 | 0.022* | 1.127 | 1.017- 1.249 | 0.022* | 0.165 |
| **Systolic blood pressure** | 325 | 0.993 | 0.979-1.007 | 0.337 | 0.993 | 0.98-1.007 | 0.341 | 0.358 |
| **Age** | 368 | 1.101 | 1.062-1.142 | <0.001* | 1.101 | 1.062-1.142 | <0.001 | 0.372 |
| **Sex (female)** | 368 | 0.961 | 0.622-1.485 | 0.858 | 0.953 | 0.617-1.472 | 0.828 | 0.131 |
| **Atrial Fibrillation** | 363 | 2.45 | 1.482-4.05 | <0.001* | 2.458 | 1.487-4.062 | <0.001* | 0.847 |
| **ApoB** | 325 | 1.000 | 0.999-1.001 | 0.673 | 1. | 0.999-1.001 | 0.674 | 0.26 |
| **ApoA1** | 325 | 0.998 | 0.997-0.999 | <0.001* | 0.998 | 0.997-0.999 | <0.001* | 0.104 |
| **ApoB/ApoA1** | 325 | 2.626 | 0.828-8.327 | 0.101 | 2.623 | 0.832-8.263 | 0.1 | 0.955 |
| **Hyperlipidemia Yes/No** | 328 | 1.753 | 1.06-2.901 | 0.029* | 1.702 | 1.029-2.814 | 0.038* | 0.18 |
| **Creatinine** | 324 | 1.014 | 1.004-1.025 | 0.006* | 1.014 | 1.004-1.025 | 0.006* | 0.58 |
| **FRS** | 323 | 1.026 | 1.009-1.043 | 0.003* | 1.026 | 1.009-1.043 | 0.002* | 0.561 |

Supplemental Material

Table 1: Univariable COX regression analysis with controls, total population and interaction variable and individual risk factors. Unadjusted. Significant results are marked with *. Abbreviations: BMI=Body Mass Index, FRS=Framingham Risk Score, HR=Hazard Ration, CI= Confidence Interval, VRF= Vascular Risk Factors.

| **Table 2** | **Controls** | **Univariable COX-regression, adjusted for age and sex** | | | **COX regression of total population + p-value for interaction variable (VRF*group)** | | | |
| --- | --- | --- | --- | --- | --- | --- | --- | --- |
|  | N | HR | 95 % CI | p-value | HR | 95 % CI | p-value | p-value interaction variable |
| **Smoking** | 365 | 1.58 | 1.021-2.446 | 0.04* | 1.511 | 0.98-2.331 | 0.062 | 0.496 |
| **BMI** | 325 | 1.017 | 0.953-1.086 | 0.603 | 1.018 | 0.953-1.086 | 0.599 | 0.5 |
| **Diabetes diagnosis** | 337 | 1.524 | 0.854-2.719 | 0.154 | 1.567 | 0.887-2.769 | 0.121 | 0.184 |
| **P-Glucose** | 325 | 1.071 | 0.96-1.195 | 0.216 | 1.072 | 0.963-1.193 | 0.207 | 0.217 |
| **Systolic blood pressure** | 325 | 0.994 | 0.98-1.008 | 0.396 | 0.995 | 0.981-1.009 | 0.447 | 0.254 |
| **Atrial Fibrillation** | 363 | 1.78 | 1.057-2.996 | 0.03* | 1.833 | 1.1-3.056 | 0.02* | 0.453 |
| **ApoB** | 325 | 1.000 | 0.999-1.001 | 0.759 | 1.0 | 0.999-1.001 | 0.739 | 0.611 |
| **ApoA1** | 325 | 0.998 | 0.997-0.999 | <0.001* | 0.998 | 0.997-0.999 | <0.001* | 0.058 |
| **ApoB/ApoA1** | 325 | 4.269 | 1.247-14.61 | 0.021* | 3.443 | 1.029-11.525 | 0.045* | 0.44 |
| **Hyperlipidemia Yes/No** | 328 | 1.866 | 1.126-3.091 | 0.016* | 1.868 | 1.129-3.092 | 0.015* | 0.123 |
| **Creatinine** | 324 | 1.013 | 1.0-1.025 | 0.046* | 1.009 | 0.997-1.021 | 0.136 | 0.612 |

Table 2: Univariable COX regression analysis with controls, interaction variable and individual risk factors. Adjusted for age and sex. Significant results are marked with *. Abbreviations: BMI=Body Mass Index, HR=Hazard Ration, CI= Confidence Interval, VRF= Vascular Risk Factors.
